# Antioxidants reduce oxidative stress in follicular fluid of aged women undergoing IVF

**DOI:** 10.1186/s12958-016-0184-7

**Published:** 2016-09-07

**Authors:** Alice Luddi, Angela Capaldo, Riccardo Focarelli, Martina Gori, Giuseppe Morgante, Paola Piomboni, Vincenzo De Leo

**Affiliations:** 1 Department Molecular and Developmental Medicine, University of Siena, Policlinico Le Scotte Viale Bracci, Siena, 53100 Italy; 2 Life Sciences Department, University of Siena, Siena, 53100 Italy

**Keywords:** Micronutrients, In vitro fertilization, Follicular fluid, Oxidative stress, Oocyte quality

## Abstract

**Background:**

The status characterized by the imbalance between pro-oxidants and antioxidants molecules, defined as oxidative stress, has been suggested to be involved in the pathogenesis of subfertility in females. This study aims to evaluate the impact of a complete micronutrients supplementation on oxidative stress levels in follicular microenvironment as well as on in vitro fertilization (IVF) outcome.

**Methods:**

This preliminary study was conducted between January 2014 and July 2015 at the Siena University Hospital Infertility Clinic. Serum and follicular fluid were collected from infertile women aged > 39 years who underwent two in vitro fertilization cycles: in the first cycle they were treated with GnRH-antagonist protocol and gonadotropins for controlled ovarian hyperstimulation, whereas in the second cycle ovarian stimulation protocol was associated to micronutrients supplementation, starting three months earlier. Protein oxidation levels and total antioxidant capacity in serum and in follicular fluid were evaluated in IVF cycles with or without micronutrients supplementation. Differences in IVF outcome parameters were statistically evaluated.

**Results:**

Two-dimensional electrophoresis analyses demonstrated that when patients assumed micronutrients before IVF cycles, follicular fluid and serum proteins were protected from oxidative damage. Comparable results were obtained when total antioxidant capacity was measured. Moreover, the mean number of good quality oocytes retrieved when patients received micronutrients supplementation was significantly increased.

**Conclusion:**

The additional treatment with micronutrients, starting three months before IVF cycles, protects the follicular microenvironment from oxidative stress, thus increasing the number of good quality oocytes recovered at the pick up.

## Background

Recent studies on the pathophysiology of couple’s infertility have shown that oxidative stress (OS) may be one of the causative factors of female infertility [1–3]. The OS is currently defined as an increase in the level of reactive oxygen species (ROS) due to the decreased antioxidant capacity of the cells to scavenge and remove these free radicals [4]. The oxidizing species are products of normal metabolic activity that, at physiological concentration, play pivotal roles both at cellular and systemic level. Numerous protective mechanisms that prevent the formation of reactive species, or their removal before they can damage cell components, have been identified [5]. Indeed, the impairment of this physiological balance between ROS production andelimination, may result in the alteration of cellular structures and macromolecules ultimately leading to cell death [6]. The main targets of ROS are lipids, nucleic acids, carbohydrates and proteins [7]. Oxidative reactions may completely alter physical/chemical properties of proteins by proteolysis, structural modifications, aberrant combinations, oxidation of the side chains of amino acids [8]. In fact, the oxidation of the -R groups of amino acids, cause the variation of the isoelectric point, whereas the formation of intramolecular bonds and/or protein cleavage into peptide fragments induces alteration of the molecular weight [9–12]. Moreover, proteins having in their backbone cysteine and methionine, sulfur-containing amino acids, are more susceptible to the attack of ROS, since thiols may undergo reversible oxidization [11, 13].

It appears that oxidative stress plays an essential physiological role in the modulation of a full spectrum of reproductive functions, such as oocyte maturation, ovarian steroidogenesis, formation of the corpus luteum and luteolysis, fertilization, embryo development and pregnancy [2, 4, 14]. The impact of OS on the reproductive potential has been investigated by several studies focused on the microenvironment surrounding the oocyte [15–20]; all these study clearly highlighted that there is a complex relationship between ROS and antioxidants in the ovary. In particular, oxidative damage has been implicated as a causal factor in the oocytes quality. The failure of antioxidant defense mechanisms to neutralize OS, allows free radicals to alter the cellular redox state, with decreased total antioxidant capacity in follicles accompanied by poor oocyte fertilization [1, 16, 21, 22].

Numerous protective substance that counteract the formation of reactive species, have been hitherto identified [23, 24] and numerous commercial preparations containing antioxidant micronutrients have been developed. Among the others, the particular micronutrients supplement, we assessed in this study, represents a suitable preparation for its broad composition, including vitamins, folates and minerals. Vitamin E and A have been reported as potent non-enzymatic antioxidants that play a significant role in protecting the integrity of the cell membrane and in promoting reproductive function. In humans it has been shown that vitamin E reacts with free radicals transforming them in hydroperoxides, substance safely metabolized by the cell; in addition, vitamin E interacts and neutralizes three important ROS, e.g. superoxide anion, hydrogen peroxide and the hydroxyl radical [23]. Finally, vitamin E is also able to interrupt the process of lipid peroxidation acting as a chain-breaking antioxidant.

It has been demonstrated that Vitamin A and carotenoids are able to protect the cells from superoxide radicals and, most importantly, a decrease in their serum concentration has been associated with anovulation [25]. Also folate has been reported as effective antioxidants, so that women receiving folic acid supplementation had a better quality oocytes and a higher degree of mature oocytes compared with women who did not receive folic acid supplementation [26].

Based on these observations, this study aimed to evaluate the effectiveness of a complete micronutrients supplementation on the level of oxidative stress, both in serum and in follicular fluid (FF), of infertile women aged > 39 years who undergo assisted reproductive technologies.

## Methods

### Patients

The study was approved by Ethic Committees of Siena University. Signed informed consent was obtained from all patients who participated in the study, performed at the Center for Couple Sterility, Siena University Hospital, between January 2014 and July 2015. A total of 18 patients aged >39 years undergoing IVF/ICSI treatment were included in the study. All patients have been unable to conceive naturally for at least 1 year before entering the study. The reasons for the couples’ infertility were male factor infertility (*n* = 8), polycystic ovary syndrome (*n* = 6), tubal occlusion (*n* = 3) and endometriosis (*n* = 1). The physical and demographic characteristics of all recruited women are shown in Table 1. All patients underwent two IVF cycles within a period of 9–12 months: in the first cycle they were treated with gonadotropins for ovulation induction, while in the second cycle gonadotropin treatment was associated to micronutrients supplementation (Elevit, Bayer; 1 cp/day), starting three months earlier.

**Table 1:** Characteristics of patients who underwent the two IVF cycles, without (untreated) and with (treated) micronutrients supplementation

Variable	Untreated	Treated	*P* value
No. of patients	18		—
Age (yrs)	40.3 ± 1.2		—
Duration of infertility (months)	26.1 ± 8.2		—
Body mass index (kg/m^2^)	22.5 ± 3.0		—
Duration of stimulation (days)	11.5 ± 0.9	12.1 ± 1.4	NS
E2 peak level	2,315 ± 490	2,270 ± 515	NS

*Note*: Values are mean ± SD. *NS* not significant

### Ovulation induction

Ovarian stimulation was performed administering recombinant gonadotropins (Pergoveris, Merck-Serono, Rome, Italy) at a dose of 150–300 IU per day from the 1^nd^ or 2^rd^ day of spontaneous or induced menstruation. The dose of gonadotropins was adjusted according to ovarian response, as detected by ultrasound examination. As soon as the dominant follicle reached 14 mm in diameter, a gonadotropin-releasing hormone (GnRH) antagonist was administered daily, until the day of ovulation triggering which was obtained by HCG injection, when at least three follicles of size >18 mm were present in the ovaries. The oocyte pick-up was performed 34–36 h after the human chorionic gonadotropin (hCG) injection. Follicular fluid was aspirated and after collecting the oocytes the fluid was centrifuged for 10 min at 1500 rpm. Only follicular fluid samples with no macroscopic evidence of blood were selected. For each patient, at the day of oocyte pickup, blood serum samples for comparative analysis was collected.

### Oocyte morphology

Oocyte morphology was evaluated according to previously published criteria [27]. Dimorphisms were subdivided into intracytoplasmic and extracytoplasmic. In the first case we evaluated the presence of incorporations, refractile bodies, vacuoles, aggregation of the smooth endoplasmatic reticulum and dense granulation. The assessment of extracytoplasmic dimorphism was based on the first polar body morphology, perivitelline space size and granularity, zona pellucida defects and shape anomalies. We considered high quality oocytes those cells without any of the previously described alterations. The evaluation was carried out by two different embryologists, blinded to the study protocol.

### The FRAP assay

The total antioxidant capacity (TAC) was measured as ferric-reducing ability of follicular fluid and serum (FRAP), according to Benzie and Strain [28], by spectrophotometric quantification. Briefly, working FRAP reagent was prepared by combining in the ratio of 10:1:1 a 300 mM acetate buffer pH3.6, 10 mM 2,4,6‐tri‐(2‐pyridyl)‐1,3,5‐triazine, in 40 mM HCl and 20 mM FeCl3. A 50 μl aliquot of follicular fluid or serum was added to 1 ml of FRAP reagent and 10 min later the absorbance measurement was taken at 593 nm. As standard reference, 50 μl of the standard (FeSO4 1 mM) was added to 1 ml of FRAP reagent. All measurements were taken at room temperature with samples protected from direct sunlight.

### Follicular fluid and blood serum proteins analysis

#### Free –SH groups labeling with MPB

This assay was carried out according to a modified protocol as described by de Lamirande and Gagnon [29]. A solution of 3-N-maleimidopropionyl biocytin (MPB; Sigma Aldrich, St Louis, MO, USA) 1 mM in Tris-HCl 15 mM pH 6.8 was added to equivalent amount of FF proteins, in a final volume of 100 μl. Protein concentration was determined by Bicinchoninic Acid Kit (Sigma-Aldrich, St Louis, MO, USA) following the manufacturer’s instruction. The mixture was then heated at 95 °C for 5 min and immediately processed for 2D electrophoresis.

#### Two-dimensional electrophoresis

Two-dimensional (2D) electrophoresis was carried out as described: 30 μg of total protein were resuspended in rehydration solution and mixed with 0.2 % immobilized pH gradient buffer (IPG, GE Healthcare, Uppsala, Sweden) with a 3–11 nonlinear pH range. Samples were loaded onto Immobiline DryStrips with immobilized nonlinear pH gradient, ranging from pH 3 to 11 (GE Healthcare). Isoelectric focusing was performed as described by Görg et al. [30] and Bjellqvist et al. [31]. Isoelectric focused strips were equilibrated for 15 min with 50 mM Tris-HCl pH 6.8 containing 30 % glycerol, 6 M urea, and 2 % sodium dodecyl sulfate (SDS) and for an additional 5 min in the same solution containing 2.5 % iodoacetamide (IAA) and 0.1 % bromophenol blue, and then placed on a 6–16 % polyacrylamide linear gradient SDS gel according to Laemmli et al. [32].

#### Western blotting

For western blotting analysis, proteins were electroblotted from polyacrylamide gels to nitrocellulose according to Towbin et al. [33]. Membranes were then incubated with streptavidin conjugated to peroxidase in TBS containing 0.2 % Tween 20, with 1 % non-fat dry milk. Reactivity was detected using an Immuno-Star HRP Chemiluminescent kit (Bio-Rad Microsciences) and revealed with an XRS instrument ChemiDoc (Bio-Rad Microsciences, Hemel Hempstead, UK). Images were then processed using the Quantity One® 4.5.7 and PDQuestTM 7.4.0 softwares (Bio-Rad Microsciences) for spots identification and quantification as pixel/mm^2^.

### Statistical analysis

Statistical analysis was performed with the software Graph pad Prism 4 (GraphPad Software, San Diego, CA, USA). The standardized values of skewness and kurtosis will be used to verify the normal distribution of data. Parametric or non-parametric tests were used when appropriate. Statistical significance will be set at a value of *P* <0.05.

## Results

**Fig. 1:**
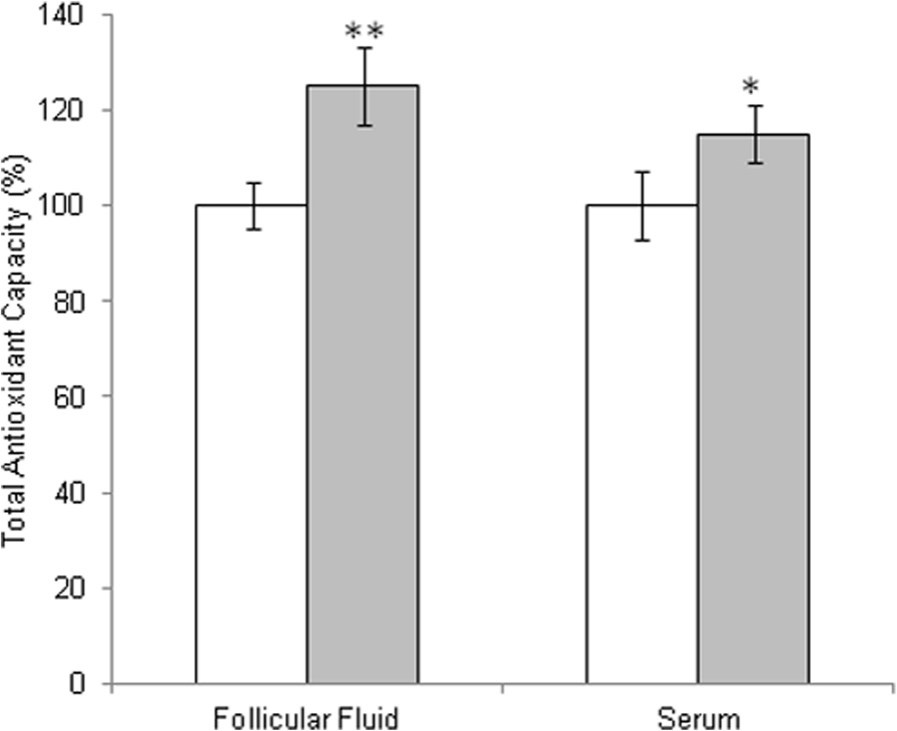
Total antioxidant capacity in follicular fluid and in serum from untreated patient (white bars) or patient treated with micronutrients supplementation (gray bars). The values are the mean of three determinations ± SD. Statistically significant difference in total antioxidant capacity is indicated (*P < 0.05)

**Fig. 2:**
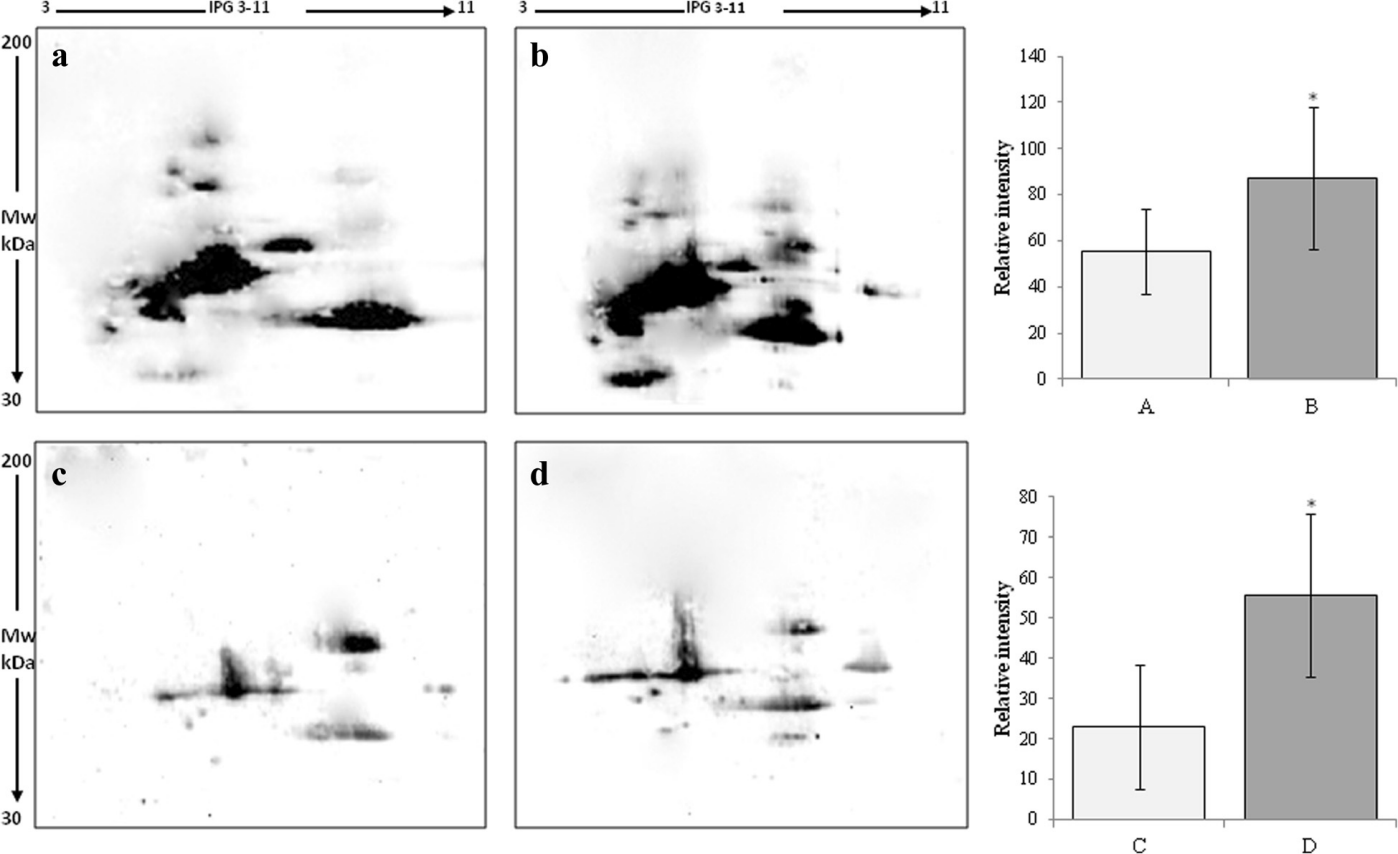
Avidin-blotting after two dimensional electrophoresis of biotin-labeled free-SH residues in serum (**a**, **b**) and follicular fluid (**c**, **d**) from untreated patient (**a**, **c**) or patient treated with micronutrients supplementation (**b**, **d**). The values are the mean ± SD. Statistically significant difference in relative intensity is indicated (*P < 0.05)

Demographic features and clinical data of patients are presented in Table 1. Total FSH-LH units, number of days of stimulation, peak E2 levels at hCG administration were not significantly different between the two IVF cycles (without or with micronutrients supplementation) in the enrolled patients. In order to detect the oxidative stress levels, we measured the total antioxidant capacity and ROS induced-damage on proteins both in follicular fluid and in serum. By means of the FRAP assay we measured total antioxidant capacity showing a significant increase in TAC both in follicular fluid and in serum of women treated with micronutrient supplementation (Fig. 1). In order to confirm these data, we analyzed all samples by two dimensional electrophoresis to monitor the presence of free-SH residues in serum and follicular fluid proteins. In Fig. 2, a representative 2D-electroforesis of serum and follicular fluid proteins is showed. By this procedure, numerous spots distributed in a pH range of about 4–7 with an apparent molecular weight ranging between 15 and 75 kDa (Fig. 2a and c) were evident in patients not receiving micronutrient supplementation (untreated) during the first IVF cycle. When the same analysis was performed in patients receiving a micronutrient supplementation in the second IVF cycle, starting from three months before controlled ovarian stimulation, we observed an increased number of proteins with free thiol groups (Fig. 2b and d). Therefore, a significant recovery of a physiological level in the overall oxidative stress status in these biological fluids after treatment is demonstrated. Indeed, both in blood serum and follicular fluid of treated women, 2D electrophoresis detected numerous spots distributed between pH 4 and 7, with a wide molecular weight ranging from about 60 to 200 kDa (Fig. 2b and d). The quantitative analysis of main spots enabled us to highlight a statistically significant increase in spot intensity in both serum and follicular fluid recovered from women treated with micronutrient supplementation (*p* < 0.05) in comparison to untreated (Fig. 2). As regard to IVF outcome parameters, we observed that the mean number of retrieved oocytes did not differ between the two cycles, whereas the mean number of oocytes not suitable for injection was significantly reduced (1.20 ± 0.77 vs. 1.88 ± 1.01; *P* = 0.01) in the micronutrients supplemented cycles. Fertilization and cleavage rates as well as the mean number of top-quality transferred embryos did not differ significantly (Table 2). Nevertheless, in the treated group, we registered a total of 3 ongoing pregnancies (pregnancy rate =17.7 %), which represent an encouraging results in aged women undergoing to IVF.

**Table 2:** Oocyte maturity and embryo score in the same patients who didn’t received (Untreated) or received (Treated) micronutrients supplementation form three months

Characteristic	Untreated	Treated	*P* value
No. of retrieved oocytes	6.76 ± 4.12	6.37 ± 3.31	NS
No. of MII oocytes	5.14 ± 3.49	4.07 ± 3.04	NS
No. of not suitable oocytes (GV-DEG)	1.88 ± 1.01	1.2 ± 0.77	0.01
Fertilization rate	0.69 ± 0,15	0.71 ± 0,28	NS
Cleavage rate	0.84 ± 0.04	0.85 ± 0.07	NS
No. of embryos transfered	2.01 ± 0.55	1.96 ± 0.75	NS
Embryo score grade 1 (%)	0.96 ± 0.5	0.91 ± 0.45	NS
Embryo score grade 2 (%)	0.87 ± 0.75	0.84 ± 0.71	NS
Embryo score grade 3 (%)	0.29 ± 0.64	0.30 ± 0.5	NS

*Note*: Values are mean ± SD. The embryos were scored according to the criteria established by Ebner et al. [27]. *DEG* degenerated occytes, *MII* metaphase II, *NS* not significant; *GV* germinal vesicle

## Discussion

Data from this study suggest that patients assuming micronutrients before IVF cycles have a high level of free-SH groups in follicular fluid and serum proteins, indicating a protective activity from oxidative damages by this supplementation.

The oxidative stress and the ROS level in biological fluid may be indirectly measured by a proteomic approach, that allow the accurate evaluation of oxidative stress induced-damage on proteins [34, 35]. The main target of free radicals are proteins that undergo to oxidation of the side chains of amino acids [9, 12] where cysteine and methionine are key components in the mechanism involved in redox balance, since thiols group (−SH) may be oxidized in an almost irreversible manner [13, 36, 37]. Indeed, an inverse correlation between levels of free-SH group in proteins from biological fluids and oxidative stress may be established. By this way, we showed in both serum and follicular fluid a dramatic increase in the level of protein free -SH groups in women treated with a micronutrients supplementation, suggesting a significant decrease of the oxidative stress in the follicular microenvironment. In this study, we confirmed the efficacy of micronutrient supplementation also by means of FRAP, a sensitive and reproducible approach commonly used to monitor total antioxidant capacity in biological fluid. This strong correlation is very important since confirm our proteomic strategy as an excellent approach to study ROS levels in biological sample. It should be outlined that the direct evaluation of the oxidative stress levels in biological fluids is very difficult because the cellular elements that produce them are absent, and because the radical species are generated and dissolve continuously. The demonstrated close relation between FRAP results and protein free -SH groups in biological samples will allow the researchers to specifically evaluate the effect of ROS by indirectly measuring the protein damage levels.

The supplementation seems to be effective not only in diminishing oxidative stress, but also in increasing oocyte quality; in fact, a significant decrease in the mean number of oocytes not suitable for injection could be observed. Our data confirmed numerous publications reporting the antioxidant capability of several micronutrients that, accordingly, are regarded as potent antioxidants. It has been reported that folates effectively scavenge oxidizing free radicals by inhibiting lipid peroxidation [34], thus playing a key role in oocyte quality and maturation [35]. In this regard, poor folate status seems to be detrimental also due to its involvement in cell division (e.g. of oogonia and/or granulosa cells), inflammatory cytokine production and defective methylation reactions [38]. The latter, in turn, are the main regulators of DNA synthesis, a pivotal process in oocytes development. It has been reported that several enzymes involved in DNA synthesis are vitamin- or zinc-dependent [39]. Zinc might also affect female fertility due to its antioxidant properties: in fact, zinc can counteract the oxidation by binding sulphydryl groups in proteins and by occupying binding sites for iron and copper in lipids, proteins and DNA [40, 41]. In this regard, the supplementation of a chelated form of zinc may contribute to the reduction of oxidative stress, while retaining safety since assumed at dose far below the tolerable upper level of 40 mg/day. Also the potential toxicity of cationes, such as iron and calcium, is hindered by their low dose. Moreover, it is remarkable that women with normal menstrual cycles could have deficiency of such compounds. An influence of these mineral on oocyte development and fertilization process has been highlighted. It has been reported that the consumption of iron supplements and non-heme iron from foods may decrease the risk of ovulatory infertility [41], and that oxidative stress in unfertilized mouse oocytes induces abnormal calcium oscillation [42].

Also vitamins seems to play a key role in oocyte maturation, since their deficiencies have been linked to a diminished fertility in animals [43]. In a recent study, we have demonstrated that vitamin receptor VDR/RXR-α (vitamin D receptor and retinoid X receptor-alpha heterodimer) are involved in follicular fluid homeostasis, suggesting the existence of a close functional correlation among follicular protein and micronutrients [44]. VDR has been hypothesized to control several human genes, thus vitamin D aberrant endogenous production or dietary up-take may severely affect the follicular physiology [42]. Vitamin D supplementation has been recently demonstrated to increase the serum soluble form of Receptor for Advanced Glycation End-products (sRAGE) [45]. sRAGE was also detected in HFF and its incremented concentration has been associated to positive IVF outcomes [46, 47]. These soluble receptors exert a “scavenger” activity of advanced glycation end-products (AGEs), potent pro-inflammatory molecules that cause ROSgeneration. The AGE/sRAGE interaction, by preventing the binding of AGE to cellular receptor (cRAGE), counterbalances the well known detrimental effects of AGE [48]. In summary, by inducing the expression of sRAGE through VDR/RXR-α, vitamin D may modulate AGE/RAGE-system, thus diminishing follicular fluid oxidative stress.

Vitamin E has been reported as a scavenger of lipid peroxyl radicals and suppresses the generation of lipid hydroperoxides from cell membrane phospholipids, by acting synergistically with other antioxidant nutrients including vitamin C, carotene, melatonin and selenium [23]. The latter is reported to act both as an antioxidant and anti-inflammatory mediator. The antioxidant activity is achieved by the reduction of lipid and hydrogen peroxides, thus lowering free radicals and ROS spreading. Moreover, selenium supplementation plays a pivotal role in regulating thyroid function by improving the conversion of plasma thyroxine (T4) into the active hormone, T3 [49].

Our data suggest that the appropriate administration of these micronutrientss may therefore have significant effects on redox status of follicular microenvironment and, ultimately, on IVF outcome.

The evidence that oocyte maturation, ovulation, luteolysis and follicle atresia are affected by ROS unbalance along with the demonstration that the oxidative stress might be influenced by micronutrients intake, supports our results reporting the effectiveness of this micronutrients supplementation also in term of oocyte quality. To our knowledge, only limited information is available on the impact of dietary micronutrients supplementation on IVF outcome.

## Conclusion

In conclusion, the biochemical evaluations carried out in the present preliminary study points out that a micronutrients supplementation may decreases oxidative stress both in serum and in follicular fluid proteins and it is positively associated with oocytes quality. These information may be helpful for clinicians in the management of women undergoing IVF treatment.

## Abbreviations

FF: Follicular fluid
GnRH: Gonadotropin-releasing hormone
hCG: Human chorionic gonadotropin
ICSI: Intra-cytoplasmic sperm injection
IVF: In vitro fertilization
OS: Oxidative stress
ROS: Reactive oxygen species
VDR/RXR-α: Vitamin D receptor and retinoid X receptor-alpha heterodimer

## References

[1] Agarwal A, Gupta S, Sharma RK (2005). Role of oxidative stress in female reproduction. Reprod Biol Endocrinol.

[2] Agarwal A, Aponte-Mellado A, Premkumar BJ (2012). The effects of oxidative stress on female reproduction: a review. Reprod Biol Endocrinol.

[3] Gupta S, Ghulmiyyah J, Sharma R, Shaman A, Gupta S (2014). Power of proteomics in linking oxidative stress and female infertility. Biomed Res Int.

[4] Ruder EH, Hartman TJ, Goldman MB (2009). Impact of oxidative stress on female fertility. Curr Opin Obstet Gynecol.

[5] Swamy M, Sirajudeen KNS, Chandran G (2009). Nitric oxide (NO), citrulline-NO cycle enzymes, glutamine synthetase, and oxidative status in kainic acid-mediated excitotoxicity in rat brain. Drug Chem Toxicol.

[6] Uttara B, Singh AV, Zamboni P, Mahajan RT (2009). Oxidative stress and neurodegenerative diseases: a review of upstream and downstream antioxidant therapeutic options. Curr Neuropharmacol.

[7] Duan J, Kasper DL (2011). Oxidative depolymerization of polysaccharides by reactive oxygen/nitrogen species. Glycobiology.

[8] Stadtman ER, Levine RL (2000). Protein oxidation. Ann N Y Acad Sci.

[9] Davies MJ, Donkor R, Dunster CA, Gee CA, Jonas S, Willson RL (1987). Desferrioxamine (Desferal) and superoxide free radicals. Formation of an enzyme-damaging nitroxide. Biochem J.

[10] Dean RT, Fu S, Stocker R, Davies MJ (1997). Biochemistry and pathology of radical-mediated protein oxidation. Biochem J.

[11] Höhn A, Jung T, Grune T (2014). Pathophysiological importance of aggregated damaged proteins. Free Radic Biol Med.

[12] Salo DC, Pacifici RE, Lin SW, Giulivi C, Davies KJ (1990). Superoxide dismutase undergoes proteolysis and fragmentation following oxidative modification and inactivation. J Biol Chem.

[13] Berlett BS, Stadtman ER (1997). Protein oxidation in aging, disease, and oxidative stress. J Biol Chem.

[14] Sugino N, Shimamura K, Takiguchi S, Tamura H, Ono M, Nakata M, Nakamura Y, Ogino K, Uda T, Kato H (1996). Changes in activity of superoxide dismutase in the human endometrium throughout the menstrual cycle and in early pregnancy. Hum Reprod.

[15] Aitken RJ (1999). The Amoroso Lecture. The human spermatozoon--a cell in crisis?. J Reprod Fertil.

[16] Oyawoye O, Abdel Gadir A, Garner A, Constantinovici N, Perrett C, Hardiman P (2003). Antioxidants and reactive oxygen species in follicular fluid of women undergoing IVF: relationship to outcome. Hum Reprod.

[17] Pasqualotto EB, Agarwal A, Sharma RK, Izzo VM, Pinotti JA, Joshi NJ, Rose BI (2004). Effect of oxidative stress in follicular fluid on the outcome of assisted reproductive procedures. Fertil Steril.

[18] Appasamy M, Jauniaux E, Serhal P, Al-Qahtani A, Groome NP, Muttukrishna S (2008). Evaluation of the relationship between follicular fluid oxidative stress, ovarian hormones, and response to gonadotropin stimulation. Fertil Steril.

[19] Fujimoto VY, Bloom MS, Huddleston HG, Shelley WB, Ocque AJ, Browne RW (2011). Correlations of follicular fluid oxidative stress biomarkers and enzyme activities with embryo morphology parameters during in vitro fertilization. Fertil Steril.

[20] Pereira AC, Martel F (2014). Oxidative stress in pregnancy and fertility pathologies. Cell Biol Toxicol.

[21] Bedaiwy MA, Elnashar SA, Goldberg JM, Sharma R, Mascha EJ, Arrigain S, Agarwal A, Falcone T (2012). Effect of follicular fluid oxidative stress parameters on intracytoplasmic sperm injection outcome. Gynecol Endocrinol.

[22] Palini S, Benedetti S, Tagliamonte MC, De Stefani S, Primiterra M, Polli V, Rocchi P, Catalani S, Battistelli S, Canestrari F, Bulletti C (2014). Influence of ovarian stimulation for IVF/ICSI on the antioxidant defence system and relationship to outcome. Reprod Biomed Online.

[23] Pham-Huy LA, He H, Pham-Huy C (2008). Free radicals, antioxidants in disease and health. Int J Biomed Sci.

[24] Noctor G, Lelarge-Trouverie C, Mhamdi A (2014). The metabolomics of oxidative stress. Phytochemistry.

[25] Al-Azemi MK, Omu AE, Fatinikun T, Mannazhath N, Abraham S (2009). Factors contributing to gender differences in serum retinol and alpha-tocopherol in infertile couples. Reprod Biomed Online.

[26] Szymański W, Kazdepka-Ziemińska A (2003). Effect of homocysteine concentration in follicular fluid on a degree of oocyte maturity. Ginekol Pol.

[27] Ebner T, Moser M, Sommergruber M, Tews G. Selection based on morphological assessment of oocytes and embryos at different stages of preimplantation development: a review. Hum Reprod Update. 2003;9:251–62. 10.1093/humupd/dmg021 12859046

[28] Benzie IF, Strain JJ (1996). The ferric reducing ability of plasma (FRAP) as a measure of “antioxidant power”: the FRAP assay. Anal Biochem.

[29] de Lamirande E, Gagnon C (1998). Paradoxical effect of reagents for sulfhydryl and disulfide groups on human sperm capacitation and superoxide production. Free Radic Biol Med.

[30] Görg A, Postel W, Günther S (1988). The current state of two-dimensional electrophoresis with immobilized pH gradients. Electrophoresis.

[31] Bjellqvist B, Pasquali C, Ravier F, Sanchez JC, Hochstrasser D (1993). A nonlinear wide-range immobilized pH gradient for two-dimensional electrophoresis and its definition in a relevant pH scale. Electrophoresis.

[32] Laemmli UK (1970). Cleavage of structural proteins during the assembly of the head of bacteriophage T4. Nature.

[33] Towbin H, Staehelin T, Gordon J (1979). Electrophoretic transfer of proteins from polyacrylamide gels to nitrocellulose sheets: procedure and some applications. Proc Natl Acad Sci U S A.

[34] Joshi R, Adhikari S, Patro BS, Chattopadhyay S, Mukherjee T (2001). Free radical scavenging behavior of folic acid: evidence for possible antioxidant activity. Free Radic Biol Med.

[35] Ebisch IMW, Thomas CMG, Peters WHM, Braat DD, Steegers-Theunissen RP (2007). The importance of folate, zinc and antioxidants in the pathogenesis and prevention of subfertility. Hum Reprod Update.

[36] Eaton S (2006). The biochemical basis of antioxidant therapy in critical illness. Proc Nutr Soc.

[37] Piomboni P, Stendardi A, Gambera L, Tatone C, Coppola L, De Leo V, Focarelli R (2012). Protein modification as oxidative stress marker in normal and pathological human seminal plasma. Redox Rep.

[38] Forges T, Monnier-Barbarino P, Alberto JM, Guéant-Rodriguez RM, Daval JL, Guéant JL (2007). Impact of folate and homocysteine metabolism on human reproductive health. Hum Reprod Update.

[39] Bray TM, Bettger WJ (1990). The physiological role of zinc as an antioxidant. Free Radic Biol Med.

[40] Tamura H, Takasaki A, Miwa I, Taniguchi K, Maekawa R, Asada H, Taketani T, Matsuoka A, Yamagata Y, Shimamura K, Morioka H, Ishikawa H, Reiter RJ, Sugino N (2008). Oxidative stress impairs oocyte quality and melatonin protects oocytes from free radical damage and improves fertilization rate. J Pineal Res.

[41] Chavarro JE, Rich-Edwards JW, Rosner BA, Willett WC (2006). Iron intake and risk of ovulatory infertility. Obstet Gynecol.

[42] Takahashi T, Takahashi E, Igarashi H, Tezuka N, Kurachi H (2003). Impact of oxidative stress in aged mouse oocytes on calcium oscillations at fertilization. Mol Reprod Dev.

[43] Lerchbaum E, Obermayer-Pietsch B (2012). Vitamin D and fertility: a systematic review. Eur J Endocrinol.

[44] Bianchi L, Gagliardi A, Landi C, Focarelli R, De Leo V, Luddi A, Bini L, Piomboni P. Protein pathways working in human follicular fluid: the future for tailored IVF? Exp Rev Mol Med 2016, in press. 10.1017/erm.2016.4 27149979

[45] Irani M, Minkoff H, Seifer DB, Merhi Z (2014). Vitamin D increases serum levels of the soluble receptor for advanced glycation End products in women with PCOS. J Clin Endocrinol Metab.

[46] Fujii EY, Nakayama M (2010). The measurements of RAGE, VEGF, and AGEs in the plasma and follicular fluid of reproductive women: the influence of aging. Fert Steril.

[47] Bonetti TC, Borges E, Braga DP, Iaconelli A, Kleine JP, Silva ID (2013). Intrafollicular soluble receptor for advanced glycation end products (sRAGE) and embryo quality in assisted reproduction. Reprod Biomed Online.

[48] Tatone C, Amicarelli F, Carbone MC, Monteleone P, Caserta D, Marci R, Artini PG, Piomboni P, Focarelli R (2008). Cellular and molecular aspects of ovarian follicle ageing. Hum Reprod Update.

[49] Rayman MP (2000). The importance of selenium to human health. Lancet.

